# Bilateral inferior petrosal sinus sampling: validity, diagnostic accuracy in lateralization of pituitary microadenoma, and treatment in eleven patients with Cushing’s syndrome – a single-center retrospective cohort study

**DOI:** 10.1186/s12902-023-01495-z

**Published:** 2023-10-23

**Authors:** Mohammadali Tavakoli Ardakani, Soghra Rabizadeh, Amirhossein Yadegar, Fatemeh Mohammadi, Sahar Karimpour Reyhan, Reihane Qahremani, Hossein Ghanaati, Alireza Esteghamati, Manouchehr Nakhjavani

**Affiliations:** 1 Endocrinology and Metabolism Research Center (EMRC), Vali-Asr Hospital, Tehran University of Medical Sciences, Tehran, Iran; 2 Advanced Diagnostic and Interventional Radiology Research Center (ADIR), Tehran University of Medical Sciences, Tehran, Iran

**Keywords:** BIPSS, Bilateral inferior petrosal sinus sampling, Cushing’s Disease, Cushing’s syndrome, EAS

## Abstract

**Background:**

This single-center retrospective cohort study aimed to describe the findings and validity of Bilateral inferior petrosal sinus sampling (BIPSS) in the differential diagnosis of patients with ACTH-dependent Cushing’s syndrome (CS).

**Methods:**

Eleven patients underwent BIPSS due to equivocal biochemical tests and imaging results. Blood samples were taken from the right inferior petrosal sinus (IPS), left IPS, and a peripheral vein before and after stimulation with desmopressin (DDAVP). ACTH and prolactin levels were measured. The diagnosis was based on the ACTH ratio between the IPS and the peripheral vein. Also, lateralization of pituitary adenoma in patients with Cushing’s disease (CD) was predicted. No significant complications were observed with BIPSS.

**Results:**

Based on the pathology report, eight patients had CD, and three had ectopic ACTH syndrome (EAS). Unstimulated BIPSS resulted in a sensitivity of 87.5%, specificity of 100%, PPV of 100%, NPV of 75%, and accuracy of 91%. Stimulated BIPSS resulted in a sensitivity of 100%, specificity of 100%, PPV of 100%, NPV of 100%, and accuracy of 100%. However, pituitary magnetic resonance imaging (MRI) had a lower diagnostic accuracy (sensitivity:62.5%, specificity:33%, PPV:71%, NPV:25%, accuracy:54%). BIPSS accurately demonstrated pituitary adenoma lateralization in 75% of patients with CD.

**Conclusions:**

This study suggests that BIPSS may be a reliable and low-complication technique in evaluating patients with ACTH-dependent CS who had inconclusive imaging and biochemical test results. The diagnostic accuracy is improved by DDAVP stimulation. Pituitary adenoma lateralization can be predicted with the aid of BIPSS.

## Introduction

All disorders with manifestations associated with glucocorticoid excess are called Cushing’s syndrome. Exogenous corticosteroids cause most CS cases, and endogenous CS cases are rare [1, 2].

The diagnosis of Cushing’s syndrome may be complicated, particularly in cases with ambiguous clinical findings, atypical presentations, and cyclic hypercortisolemia [3–5]. The initial laboratory tests for diagnosis of CS include 24-hour urinary free cortisol (UFC), late-night salivary cortisol, and low-dose dexamethasone suppression test (DST). These tests only represent hypercortisolemia [1, 2].

Once CS is diagnosed, further evaluations are needed to identify the etiology. The first step is to measure the plasma ACTH level. A low plasma ACTH level indicates ACTH-independent CS and a high level suggests ACTH-dependent CS. Normal ACTH can also occur in ACTH-dependent CS. Almost all cases of ACTH-dependent are due to pituitary adenoma (Cushing’s disease) or EAS [1, 2, 6].

Some ectopic sources include neuroendocrine tumors, bronchial carcinoma, and pancreatic carcinoma [7, 8]. Because of the high mortality in tumors associated with EAS, it is essential to differentiate CD from EAS.

To distinguish CD from EAS, a high-dose dexamethasone suppression test (HDDST), corticotropin-releasing hormone (CRH), or DDAVP stimulation tests, or pituitary MRI is recommended [1, 2, 6, 9–12]. MRI can be equivocal in half of the patients, and only relatively large lesions (> 6 mm) detected on MRI reliably confirm the diagnosis of CD with biochemical confirmation and expected clinical symptoms [9].

Considering the relatively low sensitivity and specificity of non-invasive tests [13, 14] and the high complications of the surgery, it seems reasonable to use a test with high sensitivity and specificity and few complications before resection. BIPSS with CRH or DDAVP stimulation can be helpful for further evaluation [1, 2, 10, 15, 16]. The BIPSS procedure is the same in both stimulation methods. Due to its lower cost, availability, and comparable diagnostic accuracy, using DDAVP instead of CRH for BIPSS is an alternative [17, 18]. BIPSS has been reported to have high sensitivity and specificity and is a safe procedure when performed by experienced interventional radiologists [15, 16, 19, 20].

This case series describes the experience with BIPSS and examines the validity of BIPSS for differentiating CD from EAS in patients with ACTH-dependent CS who had ambiguous or equivocal results in non-invasive tests.

## Materials and methods

### Patients

This retrospective cohort study included 11 patients with ACTH-dependent CS who underwent BIPSS between 2018 and 2020 in a tertiary care hospital.

### Data collection

Well-trained nurses conducted anthropometric measurements, including height and weight. Standing height was measured with a portable stadiometer (rounded to the nearest 0.1 cm). Using a calibrated balance beam scale, this study measured weight in the upright position (rounded to the nearest 0.1 kg). Body mass index (BMI) was calculated by dividing weight (kg) by height squared (m2). Well-trained examiners measured blood pressure (systolic and diastolic) at the left arm in the sitting position after 5 min of rest using a calibrated mercury sphygmomanometer. The blood sample was taken, and fasting blood sugar (FBS), hemoglobin (Hb), potassium (K), and creatinine (Cr) were measured. All research was performed in accordance with the Declaration of Helsinki. Informed consent was obtained from all participants or their legal guardians.

### Biochemical tests and imaging

Patients with signs and symptoms of CS underwent screening evaluations, and confirmatory tests were performed using serum cortisol and 24-hour UFC. After confirmation of CS, ACTH was measured using an immunoradiometric assay to categorize patients into ACTH-dependent or independent groups. ACTH test was performed with SIEMENS IMMULITE 2000 device with an analytical sensitivity of 5 pg/ml (1.1 pmol/l) and CV ∼7.5%. HDDST was conducted by administering 2 mg dexamethasone every 6 h for 48 h to all patients, and then serum cortisol and 24-hour UFC were rechecked. A pituitary MRI was performed with sagittal and coronal T1- and T2-weighted images before and after the gadolinium injection.

### BIPSS procedure

After biochemical tests and imaging, an experienced interventional radiologist performed bilateral and simultaneous catheterization of the inferior petrosal sinuses. Venography was obtained to evaluate venous anatomy and catheter placement. The retrograde flow of contrast dye into the contralateral cavernous sinuses was used as a marker of adequate sampling. After the correct placement of catheters, blood samples were obtained from each of three ports (peripheral (P), left inferior petrosal sinus (IPS), and right IPS) at -15, -10, -5, and 0 min. The current study used DDAVP for stimulation. After peripheral injection of 10 micrograms of DDAVP, blood samples from these three sites were obtained at + 3, +5, + 10, and + 15 min. Three samples from these sites were also obtained to measure prolactin. Upon collection, BIPSS samples were placed in an ice-water bath. At the end of the procedure, samples were taken to the laboratory, where the plasma was separated and used for immediate measurement of ACTH. Specimens were refrigerated, centrifuged, frozen, and assayed within 24 h.

After the samples were obtained, both femoral sheaths were removed, and manual compression was used to obtain hemostasis before transferring patients to the recovery room. The whole procedure took 1–2 h. Patients underwent strict bed rest for 4 h before discharge on the same day. All BIPSS were performed without significant complications, and only hematoma at the catheterization site was observed in some patients.

### BIPSS interpretation

The ratio of IPS ACTH to peripheral ACTH level (IPS/P ACTH) for each side was calculated. Baseline sampling at minute 0 with IPS/P ≥ 2 or stimulated sampling at minute 3 with 1PS/P ≥ 3 is confirmatory for CD [1, 8]. Also, the IPS/P ratio was checked for prolactin level after DDAVP stimulation (stimulated IPS/P prolactin). A stimulated IPS/P prolactin ≥ 1.8 indicates successful catheterization, meaning the catheter is correctly placed in the IPS [21]. For further evaluation, the current study normalized the ACTH to the prolactin level by dividing stimulated IPS/P ACTH into stimulated IPS/P prolactin for each side. A normalized ACTH/prolactin IPS/P ratio ≥ 1.3 supports a pituitary ACTH source (Cushing’s disease), and a normalized ratio ≤ 0.7 an ectopic source (EAS) [22]. The values between 0.7 and 1.3 are equivocal. The inter-sinus ratio was defined as the ratio of the IPS/P ACTH level of one side with the higher level divided by the IPS/P ACTH level of the other side with the lower level, either before or after stimulation. An inter-sinus ratio ≥ 1.4 indicates lateralization to the side with a higher IPS/P ACTH level [23].

### Statistical analysis

This analysis used SPSS software version 18 (SPSS, Inc.) to perform analyses. Data were expressed as numbers and percentages. Continuous variables were presented as means (± SD). This study reported the median or range when the data did not follow a normal distribution. The Shapiro-Wilk test was used to test for normality. The nonparametric Mann-Whitney U Test was utilized to compare variables. The sensitivity, specificity, positive predictive value (PPV), negative predictive value (NPV), and accuracy of the tests were calculated based on standard statistical equations.

## Results

### Baseline characteristics and clinical manifestations

This retrospective research studied 11 patients with ACTH-dependent CS, including eight females (72.7%) and three (27.3%) males. The median (Q1-Q3) age was 32.0 (22–45) years. The median (Q1-Q3) of BMI, systolic blood pressure (SBP), diastolic blood pressure (DBP), FBS, Hb, K, and Cr were 29.2 (24.8–33.3), 130.0 (125–140), 80.0 (80–95), 98.0 (88–103), 13.5 (12.4–13.9), 4.2 (3.9–4.5), and 1.0 (0.9–1.1), respectively. The demographic characteristics of patients are presented in Table 1. The Hb levels were not different in women and men (median 13.35 vs. 13.70, p-value = 0.776). In addition, no statistical difference between patients with a final diagnosis of CD and EAS was detected for Hb levels (Total: median 13.60 vs. 13.2, p-value > 0.05) (Women: median 13.5 vs. 13.2, p-value > 0.05) (Men: median 13.7 vs. 13.25, p-value > 0.05).

**Table 1:** Demographic characteristics of the studied patients

Case number	Sex	Age (years)	BMI (Kg/m^2^)	SBP (mmHg)	DBP (mmHg)	FBS (mg/dL)	Hb (g/dL)	K (mmol/L)	Cr (mg/dL)
1	F	32	36.8	125	80	103	12.4	4.1	1
2	F	27	26.2	140	98	88	12	4.2	0.8
3	F	19	26.9	130	80	98	12	4.2	0.7
4	F	21	29.2	180	110	110	13.5	2.9	0.9
5	F	35	33.3	130	80	103	15.6	3.2	1
6	F	22	21.6	128	88	73	13.8	4.5	1
7	M	51	23.5	135	89	127	13.7	4.4	1.1
8	F	24	24.8	110	70	98	16.2	4.2	1
9	M	38	35.7	145	95	99	13.9	5	1.3
10	M	45	31.9	140	80	88	12.6	4.5	1.1
11	F	47	30.1	110	70	84	13.2	3.9	0.9

BMI: body mass index; SBP: systolic blood pressure; DBP: diastolic blood pressure; FBS: fast blood sugar; Hb: hemoglobin; K: potassium; Cr: creatinine; F: female; M: male

90% of patients had at least one skin manifestation, such as striae, easy bruising, acne, hyperpigmentation, hirsutism, hair loss, edema, and hypertrichosis. Other symptoms were hypertension (HTN) (81%), reproductive dysfunction (81%), including infertility, oligomenorrhea, loss of libido, weight gain (72%), proximal muscle weakness (45%), and headache (27%) (Table 2).

**Table 2:** Clinical manifestations of the studied patients

Case number	Lag Period (months)*	Manifestation
1	6	weight gain, oligomenorrhea, hirsutism, striae, HTN, headache
2	5	weight gain, oligomenorrhea, acne, striae, HTN
3	8	weight gain, acne, hirsutism, striae, proximal muscle weakness, HTN, headache
4	6	weight gain, oligomenorrhea, infertility, hair loss, acne, hirsutism, striae, HTN
5	4	weight gain, oligomenorrhea, acne, striae, mild edema, HTN
6	12	oligomenorrhea, hirsutism, easy bruising, proximal muscle weakness, HTN
7	8	loss of libido, HTN, headache
8	12	oligomenorrhea, hirsutism, striae, proximal muscle weakness
9	4	weight gain, impotency, Hyper pigmentation, striae, acne, easy bruising, HTN
10	12	weight gain, facial edema, bone pain, proximal muscle weakness, HTN
11	3	weight gain, oligomenorrhea, hirsutism, hyper pigmentation, proximal muscle weakness

*Lag period: the gap between the onset of clinical symptoms and confirmation of the disease; HTN: hypertension

### Results of biochemical tests

Biochemical tests results, including basal serum cortisol (median:26 mcg/dl, range:15-54.5 mcg/dl), basal 24-hour UFC (median:670 mcg/dl, range:422–1545 mcg/dl), ACTH (median:58.8 pg/ml, range:25–155 pg/ml), serum cortisol after HDDST (median:14.2 mcg/dl, range:2.63-36.0 mcg/dl), 24-hour UFC after HDDST (median:292 mcg/dl, range:29.5–581 mcg/dl) are presented in Table 3. According to the basal serum cortisol results, eight patients (Cases 1, 3, 5, 7, 8, 9, 10, and 11) had basal serum cortisol levels > 22 mcg/dl, which indicates hypercortisolemia. Other patients (Cases 2, 4, and 6) had basal serum cortisol in the normal range (5–25 mcg/dl) and were considered as false negative results of this test.

**Table 3:** The results of biochemical tests in the studied patients

Case number	Basal serum cortisol (mcg/dl)	Basal 24-hour UFC (mcg/dl)	ACTH (pg/ml)	Serum cortisol after HDDST (mcg/dl) (% of Suppression)	24-hour UFC after HDDST (mcg/dl) (% of Suppression)
1	54.5	1350	53	NA	325(76%)
2	19.4	650	29.3	2.63(86%)	29.5(95%)
3	24.9	570	25	9.8(61%)	120(79%)
4	15	422	30	8.8(41%)	38(91%)
5	43.6	610	155	31.9(27%)	500(18%)
6	19.2	574	98	18.6(3%)	274(52%)
7	27.3	1100	45.8	19.5(29%)	310(72%)
8	27	670	58.8	5.8(79%)	NA
9	26	1545	82.6	23(12%)	581(62%)
10	23	1280	125	5.7(75%)	130(90%)
11	27	940	64	36(0%)	579(38%)

UFC: urine free cortisol; ACTH: adrenocorticotropic hormone; HDDST: high dose dexamethasone suppression test; NA: not assessed due to non-standard condition of the sample

All patients had elevated basal 24-hour UFC levels (422–1545 mcg/dl), indicative of hypercortisolemia (Table 3).

There were six patients with elevated peripheral ACTH levels (> 58 pg/ml) (cases 5, 6, 8, 9, 10, and 11). Other patients had ACTH within the normal range (6–58 pg/ml) (cases 1, 2, 3, 4, 7) (Table 3).

None of the patients showed suppression after 1 mg DST. After HDDST, cases 2, 3, 8, and 10 had more than 50% suppression of serum cortisol. In the other six patients, serum cortisol was not suppressed or suppressed by less than 50%. In one patient, serum cortisol levels were not measured (case 1) because the sample was not stored under standard test conditions.

Also, eight patients had more than 50% 24-hour UFC suppression after HDDST (cases 1, 2, 3, 4, 6, 7, 9, and 10). In two patients, 24-hour UFC was suppressed less than 50% (cases 5 and 11), and in one patient (case 8), the 24-hour UFC sample was not tested due to the non-standard condition of the sample.

### BIPSS results

BIPSS results before and after stimulation are shown in Table 4. The baseline value (sampling at minute 0) of IPS/P ACTH ≥ 2 confirms CD. According to this ratio, cases 1,3,4,5,6,7, and 8 were diagnosed as CD. The unilateral source for CD was confirmed in cases 1, 3, 7, and 8. BIPPS didn’t demonstrate lateralization in cases 4, 5, and 6.

**Table 4:** Baseline and stimulated IPS/P ratio for ACTH and Prolactin in the studied patients

Case Number	Diagnosis	Baseline IPS/P ACTH (≥ 2)		Stimulated IPS/P ACTH (≥ 3)		Stimulated IPS/P prolactin (≥ 1.8)		IPS/P ACTH / IPS/P prolactin (≥ 1.3)		Inter-sinus ratio (≥ 1.4)		lateralization
		**R**	**L**	**R**	**L**	**R**	**L**	**R**	**L**	Baseline	stimulated	
1	CD	**5.45**	1.15	**14.81**	1.50	**2.04**	**1.80**	**7.26**	0.83	**4.74**	**9.87**	**Right**
2	CD	0.45	1.44	1.11	**4.52**	**2.68**	**1.85**	0.41	**2.44**	**3.20**	**4.07**	**Left**
3	CD	**20.14**	1.08	**20.27**	0.97	**6.24**	**1.83**	**3.25**	0.53	**18.65**	**20.90**	**Right**
4	CD	**3.71**	**3.37**	**73.36**	**14**	**4.82**	**5.65**	**15.22**	**2.48**	1.10	**5.24**	**Right**
5	CD	**11.21**	**5.86**	**36.98**	**6.51**	**2.96**	**3.2**	**12.49**	**2.03**	**1.91**	**5.68**	**Right**
6	CD	**6.52**	**23.54**	**13.81**	**24.86**	**4.23**	**3.92**	**3.26**	**6.34**	**3.61**	**1.80**	**Left**
7	Recurrent CD	**4.77**	1.11	**9.33**	1.15	**2.02**	**1.95**	**4.62**	0.59	**4.30**	**8.11**	**Right**
8	Recurrent CD	1.48	**24.7**	**4.08**	**24**	**7.8**	**2.44**	0.52	**9.84**	**16.69**	**5.88**	**Left**
9	EAS	0.96	0.87	1.27	1.18	**2.29**	**9.21**	0.55	0.13	1.10	1.08	Equivocal
10	EAS	1.16	1.13	1.40	1.37	**5.28**	**5.09**	0.26	0.27	1.03	1.02	Equivocal
11	EAS	1.21	1.16	1.40	1.27	**3.43**	**3.43**	0.41	0.37	1.04	1.10	Equivocal

IPS: inferior petrosal sinus; P: peripheral; ACTH: adrenocorticotropic hormone; CD: Cushing’s disease; EAS: ectopic ACTH syndrome

The highest IPS/P ACTH ratio was 3 min after the DDAVP injection. A sampling at minute 3 with stimulated IPS/P ACTH ≥ 3 confirms CD. This ratio confirmed CD in cases 1–8 and showed a unilateral source for CD in cases 1, 2, 3, and 7. The ratio didn’t demonstrate lateralization in cases 4, 5, 6, and 8. The stimulated IPS/P prolactin was ≥ 1.8 in all cases.

**Fig. 1:**
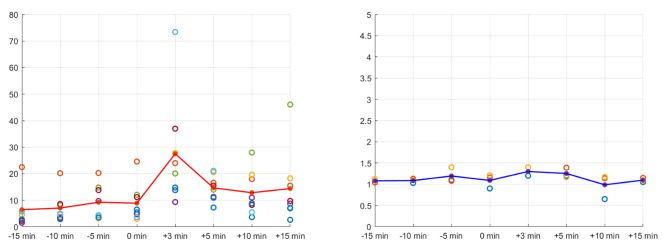
Comparison of mean values of IPS/P ACTH in CD (Lt.) and EAS (Rt.). IPS; inferior petrosal sinus; P: peripheral; ACTH: adrenocorticotropic hormone; CD: Cushing’s disease; EAS: ectopic ACTH syndrome; Lt: left; Rt: right

The variability in the IPS/P ACTH ratio in patients with CD is shown in Fig. 1. The peak of this ratio was 3 min after the DDAVP injection. In patients with EAS, there were no changes before or after the DDAVP stimulation.

According to the Prolactin-normalized ACTH IPS/P ratios, eight patients (cases 1–8) were diagnosed as CD and three as EAS (cases 9–11). In cases 1, 2, 3, 7, and 8, unilateral sources of CD were confirmed, but in cases 4,5 and 6, bilateral sources were detected (Table 4).

According to the inter-sinus ratio, BIPSS could lateralize the source of ACTH in all patients with CD. The inter-sinus ratio in patients with EAS could not lateralize any pituitary source for ACTH (Table 4).

In five patients with CD and one with EAS, the highest peripheral ACTH level was observed 15 min after stimulation. Two patients with CD and one with EAS had the highest peripheral ACTH level 10 min after stimulation. Only one patient with CD and one with EAS had the highest peripheral ACTH level 5 min after stimulation. No patient had maximum peripheral ACTH levels in the first post-stimulation sample (minute 3).

The larger numerator or smaller denominator produces a higher value in a ratio. In the samples obtained immediately after stimulation, the highest concentration of ACTH was in the IPS, and the lowest was in the peripheral blood. Therefore, as mentioned, the highest post-stimulation value of the IPS/P ACTH ratio was obtained at minute 3.

### MRI results

MRI results showed pituitary adenoma in five patients, enhancement in one patient, pituitary mass and lesion in two patients, empty sella in two patients, and possible pituitary adenoma and adrenal mass in one patient (Table 5).

**Table 5:** Final diagnosis, lateralization, MRI results, and management

Case number	Diagnosis	MRI	Lateralization according to MRI	Lateralization according to inter-sinus ratio	Lateralization at the time of surgery	IHC	Management
1	CD	3 mm Rt. adenoma	Rt.	Rt.	Rt.	ACTH + pituitary adenoma	TSS
2	CD	5.5 mm Rt. and 2.5 mm Lt. adenoma	Bilateral	Lt.	Bilateral	ACTH + pituitary adenoma	TSS
3	CD	3.8 mm Rt. adenoma	Rt.	Rt.	Rt.	ACTH + pituitary adenoma	TSS
4	CD	Lt. side enhancement	Lt.	Rt.	Rt.	corticotroph adenoma	TSS
5	CD	6 mm Rt. adenoma and 4.5 mm LLL nodule	Bilateral	Rt.	Rt.	ACTH + pituitary adenoma	TSS
6	CD	4 mm Lt. adenoma and 4 mm RUL nodule	Bilateral	Lt.	Lt.	ACTH + pituitary adenoma	TSS
7	recurrent CD	13 mm Rt. low signal pituitary Mass	Rt.	Rt	Rt.	ACTH + pituitary adenoma	TSS
8	recurrent CD	partially empty sella	Equivocal	Lt.		ACTH - Pituitary Mass	TSS
9	EAS	empty sella	Equivocal	Equivocal			Medical treatment
10	EAS	1.9 mm Rt. pituitary Lesion & LLL mass	Bilateral	Equivocal		ACTH + carcinoid tumor of the lung	Lung lobectomy
11	EAS	possible pituitary adenoma and adrenal mass	Equivocal	Equivocal		Pheochromocytoma	bilateral adrenalectomy

IHC: immunohistochemistry; CD: Cushing’s disease; EAS: ectopic ACTH syndrome; Rt: right; Lt: left; ACTH: adrenocorticotropic hormone; TSS: transsphenoidal surgery; LLL: left lower lobe; RUL: right upper lobe

### Immunohistochemistry (IHC) results

According to the pathology report, eight patients were confirmed as CD (Table 5). The other two patients were EAS (one carcinoid tumor of the lung and one pheochromocytoma). One patient had no documented pathologic source of hypercortisolemia because the patient did not consent to surgery, and the diagnosis of EAS was made based on the results of biochemical tests.

### BIPSS vs. MRI results

MRI results showed pituitary adenoma in five patients with CD. MRI and BIPSS showed the adenoma on a similar side in two of them. In the other three patients, MRI showed bilateral adenoma, but BIPSS lateralized the adenoma to one side. One of the other three patients had only left-sided enhancement but no overt adenoma on MRI, whereas BIPSS lateralized the adenoma to the right side. One patient had a low-signal pituitary mass on the right side on MRI, and BIPSS also lateralized to the right. Another patient with a history of transsphenoidal surgery (TSS), diagnosed as recurrent CD, had a partially empty sella. MRI was equivocal, but BIPSS lateralized to the left side.

Among patients with EAS, one with an equivocal BIPSS result had an empty sella on MRI. Two other patients had pituitary lesions on MRI, but BIPSS results were equivocal.

### Comparison between BIPSS, MRI, and surgery

Among patients with CD, the final diagnosis based on surgery in three patients was consistent with MRI and BIPSS results and lateralized the adenoma on the same side. In one patient, the surgery result was similar to the MRI findings and showed bilateral adenoma, but BIPSS showed adenoma on the left side. In the patient with equivocal MRI findings and a history of TSS, IHC could not identify ACTH +, although BIPSS lateralized to the left side. In three other patients, surgery results were concordant with BIPSS and lateralized the adenoma on the same side, although MRI showed discordant results.

### Validity of BIPSS

Baseline IPS/P ACTH resulted in a sensitivity of 87.5%, specificity of 100%, PPV of 100%, NPV of 75%, and accuracy of 91%. Stimulation with DDAVP improved validity. Both stimulated IPS/P ACTH and normalized ACTH/prolactin IPS/P ratio resulted in a sensitivity of 100%, specificity of 100%, PPV of 100%, NPV of 100%, and accuracy of 100%. BIPSS, either unstimulated or stimulated, had higher validity than MRI, with a sensitivity of 62.5%, specificity of 33%, PPV of 71%, NPV of 25%, and accuracy of 54%. BIPSS accurately predicted pituitary adenoma lateralization in 75% of patients with CD.

## Discussion

In this study, BIPSS before stimulation showed a sensitivity of 87.5% and a specificity of 100%. However, BIPSS after stimulation showed a sensitivity of 100% and specificity of 100%. It has been demonstrated that the sensitivity of BIPSS can vary from 88 to 100%, and its specificity from 67 to 100% in the diagnosis of CD [24]. Previous studies have reported sensitivity and specificity of more than 80% and 90% for BIPSS, and the combination of BIPSS with stimulation by CRH or DDAVP improves the sensitivity and specificity to more than 95 and 100%, respectively [15, 19, 25]. Chen et al. suggested the optimal IPS:P cutoff value of 1.4 before and 2.8 after stimulation [20]. Considering these cutoffs, the only patient in this study who was negative for CD before stimulation becomes positive, and the sensitivity before stimulation increases from 87.5 to 100%. The diagnostic accuracy after stimulation remains unchanged. Results of the current study showed that BIPSS is highly valued in final diagnosis, even without stimulation.

In this investigation, the utilization of Prolactin-normalized ACTH IPS/P ratios exhibited a sensitivity and specificity of 100% for the CD diagnosis. This finding aligns with research conducted by Detomas et al., which reported a sensitivity of 96% and specificity of 100% for the normalized ACTH: Prolactin IPS/P ratio [26]. It seems that concurrently assessing prolactin levels may potentially enhance the diagnostic accuracy of BIPSS. However, the current literature is inconsistent. Some studies do not support the use of prolactin to diagnose CD [27].

In all patients, the IPS/P ACTH ratio at minute 15 did not show a considerable difference from this ratio at minute 0. Previous studies have shown that sampling at minute 15 is not helpful for diagnosis [1, 15, 20, 28]. Unlike the IPS/P ACTH ratio, six patients had the highest peripheral ACTH level at minute 15 after stimulation, but no patient had it at minute 3 after stimulation. However, more studies are needed to obtain more precise results, and this study’s sample size was limited.

BIPSS accurately lateralized the adenoma in six patients with CD, but MRI was able to lateralize the adenoma in two patients correctly. BIPSS had higher validity than MRI in differentiating CD from EAS, both with and without stimulation. The current literature is controversial. Colao et al. reported that adenoma could be accurately localized in 65% of patients using IPSS [23]. However, Lefournier et al. showed that the diagnostic accuracy of IPSS in identifying the side of the pituitary adenoma was 57% [28]. Wind et al. showed that the PPV for IPSS to identify the tumor side correctly was 69%. Additionally, MRI was more accurate than IPSS in tumor lateralization [29]. Earlier studies have shown that MRI may show a pituitary lesion, and BIPSS indicates a pituitary adenoma. However, the lesion observed on the MRI is not related to the pituitary adenoma [1, 15, 19, 25, 28]. Also, MRI may show pituitary lesions, while BIPSS indicates EAS.

In the current study, the concordance of IHC results with BIPSS and MRI findings was inconclusive, possibly due to the limited number of patients. However, there is disagreement about the role of pathological study in diagnosis [19, 28].

Eight patients had elevated basal serum cortisol levels in this study (Sensitivity:73%). Instead, all patients had hypercortisolemia according to basal 24-hour UFC results, and no false-negative results were observed (Sensitivity:100%). This study’s findings were consistent with previous studies regarding low sensitivity for basal serum cortisol and high sensitivity for 24-hour UFC as screening tests for hypercortisolemia [6, 30, 31].

After HDDST, basal serum cortisol suppression was observed in three patients with CD (cases 2, 3, and 8) but not in the others with CD. Also, serum cortisol levels were suppressed after HDDST in a patient with EAS who had a lung carcinoid tumor. Arnaldi et al. showed that some carcinoid tumors might be sensitive to HDDST, and suppression of serum cortisol may be observed after this test [1, 32]. After HDDST, six patients with CD had suppressed 24-hour UFC, but one did not show more than 50% suppression. Two patients with EAS had more than 50% 24-hour UFC suppression.

According to the final pathology report, the sensitivity of serum and urine cortisol level tests after HDDST was 43% and 86%, and the specificity was 67% and 33%, respectively. PPV in both was 75%, NPV was 33% and 50%, and accuracy was 50% and 70%, respectively, which shows that these preliminary tests cannot be a good guide for the final diagnosis and subsequent treatment planning. Previous studies showed that more than one biochemical test could improve the accuracy for differentiating between CD and EAS [1, 5, 6, 9, 31]. The current study confirms the importance of using more than one biochemical test for diagnosing hypercortisolemia and diagnosing CD from EAS.

Detomas et al. reported that Hb levels were high in females with CS while they were low in males with CS. Furthermore, there were lower levels of Hb in EAS than in CD in females [33]. In the current study, the Hb levels were not different in women and men. Furthermore, no statistical difference was observed for Hb levels between patients with a final diagnosis of CD and EAS. Hb levels did not contribute to diagnosing ACTH-dependent CS in this analysis.

There were some limitations in this study. First, the sample size was relatively small. Second, it was a retrospective study. Further studies could investigate the BIPSS in a larger sample size and determine the validity of this method in patients with CS.

## Conclusions

The current study suggests that BIPSS can be a reliable and low-complication method in evaluating patients with ACTH-dependent CS who had equivocal results in imaging and biochemical tests, even before stimulation. Stimulation with DDAVP increases diagnostic accuracy. BIPSS can be used to predict the lateralization of the pituitary adenoma.

## Data Availability

All data generated or analyzed during this study are included in this published article.

## Abbreviations

BIPSS: Bilateral inferior petrosal sinus sampling
ACTH: Adrenocorticotropic hormone
CS: Cushing’s syndrome
IPS: Inferior petrosal sinus
DDAVP: Desmopressin
CD: Cushing’s disease
EAS: Ectopic ACTH syndrome
MRI: Magnetic resonance imaging
UFC: Urinary free cortisol
DST: Dexamethasone suppression test
HDDST: High-dose dexamethasone suppression test
CRH: Corticotropin-releasing hormone
BMI: Body mass index
FBS: Fasting blood glucose
Hb: Hemoglobin
Cr: Creatinine
PPV: Positive predictive value
NPV: Negative predictive value
SBP: Systolic blood pressure
DBP: Diastolic blood pressure
K: Potassium
HTN: Hypertension
IHC: Immunohistochemistry
TSS: Transsphenoidal surgery
